# Petrolina

**Published:** 1881-09-20

**Authors:** 


*Petrolina.*—Among the best of the modern additions to the armamentarium of the practitioner petrolina may be numbered. Bland, soothing and unirritating, it is adapted to all conditions wherein cerates or ointments are indicated. As prepared by the Binghamton Oil Refining Company it is a beautiful and excellent preparation, and is made the base of a number of valuable combinations in the shape of salves, etc. Be sure to see their advertisement.

